# Communicating *BRCA* research results to patients enrolled in international clinical trials: lessons learnt from the AGO-OVAR 16 study

**DOI:** 10.1186/s12910-016-0144-y

**Published:** 2016-10-21

**Authors:** David J. Pulford, Philipp Harter, Anne Floquet, Catherine Barrett, Dong Hoon Suh, Michael Friedlander, José Angel Arranz, Kosei Hasegawa, Hiroomi Tada, Peter Vuylsteke, Mansoor R. Mirza, Nicoletta Donadello, Giovanni Scambia, Toby Johnson, Charles Cox, John K. Chan, Martin Imhof, Thomas J. Herzog, Paula Calvert, Pauline Wimberger, Dominique Berton-Rigaud, Myong Cheol Lim, Gabriele Elser, Chun-Fang Xu, Andreas du Bois

**Affiliations:** 1 GlaxoSmithKline Medicines Research Centre, Gunnels Wood Road, Stevenage, Hertfordshire SG1 2NY UK; 2 AGO Study group and Department of Gynecology & Gynecologic Oncology, Kliniken Essen Mitte (KEM), Essen, Germany; 3 GINECO and Medical Oncology, Institut Bergonié, Bordeaux, France; 4 GSK Stockley Park West, 1-3 Iron Bridge Road, Uxbridge, UK; 5 Department of Obstetrics & Gynecology, KGOG and Seoul National University Bundang Hospital, 82, Gumi-ro 173 Beon-gil, Bundang-gu, Seongnam-si, Gyeonggi-do Korea; 6 ANZGOG and The Prince of Wales Clinical School University of New South Wales, Randwick, NSW Australia; 7 GEICO and Hospital General Universitario Gregorio Marañón, Madrid, Spain; 8 JGOG and Saitama Medical University International Medical Center, Hidaka, Japan; 9 GlaxoSmithKline Research and Development, Philadelphia, USA; 10 BGOG and Medical Oncology, Université Catholique de Louvain, CHU UCL Namur, Belgium; 11 NSGO and Rigshospitalet, Copenhagen, Denmark; 12 MaNGO and Ospedale Filippo del Ponte, Varese, Italy; 13 Department of Woman Health, MITO and Catholic University of the Sacred Heart, Rome, Italy; 14 Palo Alto Medical Foundation, San Francisco, CA USA; 15 Karl Landsteiner Research Institute and Department of Obstetrics and Gynecology, General Public Teaching Hospital, Korneuburg, Vienna, Austria; 16 NYGOG and University of Cincinnati Cancer Institute, Cincinnati, OH USA; 17 Cancer Trials Ireland, 60 Fitzwilliam Square N, Dublin 2, Ireland; 18 AGO Germany and Department of Gynecology and Obstetrics, TU Dresden, Carl-Gustav-Carus University, Dresden, Germany; 19 GINECO and ICO Centre René Gauducheau, Saint-Herblain, France; 20 KGOG, Gynecologic Cancer Branch and Center for Uterine Cancer, National Cancer Center, Goyang, Korea; 21 AGO Study Group, Wiesbaden, Germany; 22 Immuno-Oncology Development, Incyte Corporation, Wilmington, DE USA

**Keywords:** Ovarian cancer, *BRCA* mutation, Bioethics, Pharmacogenetic research, Incidental finding

## Abstract

**Background:**

The focus on translational research in clinical trials has the potential to generate clinically relevant genetic data that could have importance to patients. This raises challenging questions about communicating relevant genetic research results to individual patients.

**Methods:**

An exploratory pharmacogenetic analysis was conducted in the international ovarian cancer phase III trial, AGO-OVAR 16, which found that patients with clinically important germ-line *BRCA1/2* mutations had improved progression-free survival prognosis. Mechanisms to communicate *BRCA* results were evaluated, because these findings may be beneficial to patients and their families.

**Results:**

Communicating individual *BRCA* results was not anticipated during clinical trial design. Consequently, options were not available for patients to indicate their preference for receiving their individual results when they signed pharmacogenetic informed consent. Differences in local requirements, clinical practice, and opinion regarding the ethical aspects of how to convey genetic results to patients are all potential barriers to returning individual *BRCA* results to patients. Communicating the aggregate *BRCA* result from this study provided clinical investigators with a mechanism to disseminate the overall study finding to patients while taking individual circumstances, local guidelines and clinical practice into account.

**Conclusion:**

This study illustrates the importance of increasing the clarity and scope of informed consent and the need for patient engagement to ensure clinical trial participants can indicate their preference regarding receipt of potentially important individual pharmacogenetic results.

**Trial registration:**

This study was registered in the NCT Clinical Trial Registry under NCT00866697 on March 19, 2009, following approval from participating ethics committees (Additional file 1).

**Electronic supplementary material:**

The online version of this article (doi:10.1186/s12910-016-0144-y) contains supplementary material, which is available to authorized users.

## Background

There are numerous arguments in favor of individual genetic research results being communicated to study participants, including the right of the participants to receive potentially important research information about themselves and the possible benefit from clinically actionable findings to both participants and their families [1]. Indeed, research has shown that most research participants are interested in receiving their genomic research results [2] and that this interest extends to participants in different countries [3]. Arguments against communicating individual results include the fact that the significance of the genetic research results may be uncertain and that there may be a potential for participants to misinterpret their results or make ill-informed treatment decisions [4], unless they receive genetic counselling and their results are confirmed in clinically accredited laboratories. Many countries have ethics guidelines recognizing that exploratory genetic results may have limited clinical utility; while in some other countries regulation provides participants with the right to access their individual results [5]. In the United States, where there is no explicit legal requirement to return genetic research findings [6], the American College of Medical Genetics and Genomics suggested that pathogenic mutations in 56 specified genes should be returned to patients undergoing clinical exome and genome sequencing [7], a position that has further added to the debate on whether similar recommendations are required in the genetic research setting.

Clinical diagnostic genetic testing utilizes validated tests that are conducted in accredited laboratories, to assess genetic factors to diagnose or predict disease and/or to inform treatment options. Pharmacogenetic (PGx) research aims to understand genetic influences on the response to medicines. Most PGx analyses are exploratory, often conducted retrospectively in a non-accredited environment, and are not designed to inform the clinical care of the individual study participants. These exploratory PGx sub-studies are generally not designed with communication of individual results in mind, and international or multicenter clinical studies raise further operational challenges (for example, consent documents may differ between countries to comply with local law or practice, and PGx researchers are not in direct contact with study patients). Despite this, the increasingly routine application of genome-wide approaches in PGx research increases the probability that researchers may identify findings with a potential clinical significance to study participants and their families, raising the question of how to manage and communicate such findings.

While international policies suggest that there may be an ethical duty of care to communicate individual genetic research results if certain conditions are met [8], there is a lack of agreement in guidance between countries [9], and the potential benefits or harm of sharing non-accredited research results requires further evaluation [10]. Suggested minimum requirements for the communication of individual genetic research results require that results be validated and clinically relevant [11], where intervention has the potential to influence treatment or patient management [12]. Furthermore, the European Commission has highlighted the importance of the informed consent process and the need for full transparency on the provision of test results to individuals and populations [13]. However, the informed consent forms for most clinical trials conducted to date typically do not provide participants with the option to indicate whether or not they wish to receive their individual genetic results.

The AGO-OVAR 16 clinical trial evaluated the efficacy and safety of pazopanib versus placebo as maintenance therapy in women with FIGO Stage II-IV epithelial ovarian, fallopian tube, or primary peritoneal cancer who had not progressed after first-line chemotherapy [14]. It is well known that germ-line and somatic pathogenic mutations in the tumor suppressor genes, *BRCA1* and *BRCA2* (*BRCA1/2*), confer increased risk for ovarian and breast cancers [15]. Other studies, reported after AGO-OVAR 16 was designed, have shown that these same mutations confer increased sensitivity to platinum-based therapies and to poly ADP ribose polymerase (PARP) inhibitors [16–18]. Given this association and following a specific question from a regulatory agency, Harter and colleagues conducted a post-hoc exploratory analysis to evaluate the potential effect of *BRCA1/2* mutation on pazopanib efficacy in AGO-OVAR 16. This analysis demonstrated that 15 % of participants who could be evaluated in a PGx sub-study carried a clinically important germ-line mutation in *BRCA1* or *BRCA2* (hereafter referred to as a *BRCA* mutation), which was associated with better progression-free survival (PFS) prognosis [19]. Because germ-line pathogenic mutations in *BRCA1/2* are a strong genetic risk factor for breast and epithelial ovarian cancer [15], and are associated with disease prognosis and response to treatment [16–18], the individual research results may be regarded as medically important information for patients who do not yet have a diagnostic quality *BRCA1/2* test result. Although published opinion on the return of results is helpful, there are limited examples involving the actual return of PGx research results, and to our knowledge, of those that have been reported [20], none discuss this situation in an international clinical trial setting. Here, we discuss the considerations that led the AGO-OVAR 16 Steering Committee and pharmaceutical sponsor to develop a framework for communication of *BRCA* research results to ovarian cancer patients enrolled in AGO-OVAR 16.

## Methods

### Clinical protocol and informed consent

The AGO-OVAR 16 clinical trial (ClinicalTrials.gov Identifier: NCT00866697) has been previously described [14]. The clinical protocol and patient informed consent form (ICF) incorporated a description and objectives of exploratory PGx research. Participants included in the PGx sub-study provided additional consent and a blood sample for genetic research [19]. The clinical study was conducted in accordance with the Declaration of Helsinki; protocols and ICFs were reviewed and approved by Institutional Review Boards and Independent Ethics Committees (Additional file 1) according to local guidelines.

### Genotyping and *BRCA* variant calling

*BRCA1*/*2* exon genotyping was conducted in non-accredited research laboratories using next-generation sequencing (NGS) technologies that aimed to screen for single nucleotide variants (SNV), and small insertions and deletions. Reagents used were intended for research only and were not approved for diagnostic purposes. While terminology to describe genetic variation is emerging [21], for simplicity the term “mutation” is used to describe “pathogenic” or “likely-pathogenic” variants throughout this paper. Specifically, in this work mutations in *BRCA1* and *BRCA2* are defined as variants annotated as clinically important in the Breast Cancer Information Core (BIC) database [22]. Confidence in genotyping results was confirmed by checking concordance with genotype data from a genome-wide array platform (which assayed mostly non-clinically important variants for each sample), and by manual inspection of aligned short reads and calls when mutations were identified. *BRCA1*/*2* genotype data were available for 664 (~71 %) of 940 clinical trial participants (hereafter referred to as patients). Ninety-seven of 664 patients (15 %) were called as carrying a *BRCA1/2* mutation [19].

### Process

The pharmaceutical sponsor (GlaxoSmithKline) and the AGO-OVAR 16 Steering Committee evaluated the exploratory PGx results and identified the return of *BRCA1*/*2* results as an area for consideration. The AGO-OVAR 16 Steering Committee was made up of members of each collaborative research group involved in the study, representing 17 countries (Additional file 2), and included physicians involved in the care of patients enrolled in the clinical trial. Clinical protocol and ICF language relating to the handling of PGx data was reviewed to determine likely patient understanding regarding the return of PGx results. With genotype data generated in a non-accredited research setting, the analytical validity of individual research results was reviewed. In addition, the medical importance of available *BRCA1*/*2* data in the AGO-OVAR 16 study population was considered. Additional feedback was obtained from the sponsor’s local operating companies and collaborative groups regarding local guidelines on communication of individual genetic research results. Ultimately the approach taken was agreed to by members of the AGO-OVAR 16 Steering Committee and the sponsor. The sponsor confirmed that information sent to study sites was received by the investigators but did not determine whether investigators shared information with their patients because the investigators were not under any obligation to do so.

## Results

### Joint Sponsor and Steering Committee (JSC) considerations

The AGO-OVAR 16 PGx sub-study tested the association between germ-line *BRCA1*/*2* mutations and PFS in patients receiving pazopanib versus placebo as maintenance therapy [19]. While the lifetime cumulative risk of breast or ovarian cancer is high for *BRCA1/2* mutation carriers, the actual risk conferred by a particular mutation is difficult to estimate [23]. Nonetheless, *BRCA1* or *BRCA2* are amongst genes identified as having direct clinical utility [20]. Knowledge of individual *BRCA1*/*2* mutation status may inform disease prognosis and treatment decisions (e.g., continuation of platinum-based chemotherapies, use of PARP inhibitors, increased risk of other cancers such as breast cancer, and enrollment into future clinical trials) and may also provide important information that the patient can share with her family members. Consequently, consideration was given to whether and how to return *BRCA* research results to patients in the AGO-OVAR 16 trial, and several options were evaluated as described in Table 1.

**Table 1:** Options for communication of PGx data to trial participants

Option 1: Not to proactively communicate either the individual or aggregate research results to patients; the results will be published in scientific journals.	
Option 2: Communicate a summary of the aggregate research results to patients, and provide them with the option to receive or not receive their individual (research-quality) *BRCA* result	
Option 3: Communicate a summary of the aggregate research results and encourage patients who have not had a diagnostic-quality *BRCA* test to seek pre-test counselling and a diagnostic *BRCA* test.	

*PGx* pharmacogenetic

These options were considered in the context of the following: (a) medical relevance of the *BRCA* research results to AGO-OVAR 16 patients, (b) information contained in the study protocol and ICF, (c) ethical opinion regarding the likely expectations of patients at the time of enrollment, (d) the requirements for genetic counselling, and (e) the analytical validity and whether it is appropriate to return exploratory research data not intended for diagnostic purposes. Furthermore, an understanding of the local environment and guidelines that may influence local clinical practice in a global clinical trial were considered.

### Medical relevance of *BRCA* test results in the AGO-OVAR 16 ovarian cancer population

While the association between *BRCA* germ-line mutations and breast and ovarian cancer is well established, risk estimates for the development of these cancers vary markedly [24]. The incidence of breast cancer in *BRCA* variant carriers after a diagnosis of epithelial ovarian cancer is less than 10 % at 10 years, and overall survival is dominated by ovarian cancer-related mortality rather than subsequent breast cancer [25, 26]. The majority (>91 %) of patients in AGO-OVAR 16 had late stage (III-IV) disease [14] and, although it is difficult to predict absolute risk, our rough estimates of the proportion of patients who would have a new primary *BRCA*-associated cancer occurring in this population was <5 %. Thus, the benefit of communicating individual *BRCA1/2* research results with patients who already have ovarian cancer, with respect to the risk of developing further *BRCA-*related cancers, is unclear and did not overcome the issues described below. Patients in AGO-OVAR 16 had already developed ovarian cancer and in many cases had progressed. Nevertheless, first-degree relatives of these patients may still be healthy, and knowledge of their *BRCA* status may be very informative, as is discussed below.

Differential response to therapy by *BRCA1/2* status is another area that could be considered clinically important. Increased sensitivity to platinum-based chemotherapy was observed in *BRCA* mutation carriers [17, 18], and maintenance therapy with olaparib is most effective in *BRCA* mutation carriers who had platinum-sensitive recurrent serous ovarian cancer [16]. However, in some countries (for example, Spain) the guidance regarding the management of ovarian cancer does not include the use of *BRCA* testing [27], but may be revised following the approval of olaparib for the treatment of ovarian cancer in Europe. Nonetheless, the provision of research-grade *BRCA* test results may not be aligned with clinical practice or law in some jurisdictions, presenting an ethical dilemma that requires further consideration. Essentially, if the research results are clinically relevant and appropriate to return in one jurisdiction, it would be hard to argue that they are not relevant in another jurisdiction.

While opinion supports the return of medically actionable research results [1], clinician opinion differs on whether patients should be directly notified of their individual PGx research results [28]. Similarly, views varied significantly amongst the AGO-OVAR 16 study physicians regarding the appropriateness of communicating the individual *BRCA* research results generated in the study. However, there was overall general agreement that the *BRCA* research results may be of interest and benefit and that steps should be taken to find an appropriate mechanism to share the results with study patients.

### Protocol and informed consent

The AGO-OVAR 16 trial was initiated in 2009 and included exploratory PGx research objectives. The protocol stated that PGx research results would not be communicated unless they were known to be relevant to the “participant’s medical care at the time of the study,” because the PGx results would be preliminary and their significance and scientific validity undetermined. The ICF template (from which individual country ICFs were derived) highlighted that study information not helpful to the participant’s health-care “like most genetic research” would not be returned, although new medical information found during the study would be shared with the patients if it was considered important to their health. The ICF template also highlighted that a participant can request medical information about themselves once the study is completed. The ICF template further stated that tests on patient blood samples would aim to understand response to medicine, but would not provide information about inherited diseases or diseases that may develop in the future (and thus failed to anticipate the possibility that the same genetic variants may be associated with both response to medicine and risk of disease). Consistent with the protocol and ICF, the PGx sub-study evaluated the role of genetic variants, including *BRCA1/2* mutations, on response to treatments. Local ICFs were generally consistent with the study ICF template and collectively did not highlight that PGx research information would be proactively returned.

### Ethics opinion

The German lead ethics committee (EC) provided the opinion that patient informed consent did not cover the return of individual PGx research results, a view supported by the collaborative research groups involved in the study.

Generally, research participants should have the opportunity to decide whether they want to receive their individual results prior to their participation in the study [8, 12, 29]. However, since there was no expectation that individual PGx results would be returned, patients in AGO-OVAR 16 were not given an opportunity to indicate whether they wished to receive their results. An alternative approach could be to contact research participants after completion of the PGx study to determine whether they would like to receive individual PGx research results retrospectively; however, this could be considered coercive [10], that is, patients may feel compelled to receive their results. Indeed our discussions highlighted concerns for patients who had previously indicated they did not want to be contacted after completing the study. Re-contacting these patients may cause further alarm and, although the ethical nature of returning individual *BRCA* test results in these cases can be further debated, these concerns highlighted the difficulties of satisfying all scenarios.

### Genetic counselling

The European Society of Medical Oncology (ESMO) recommends that clinical *BRCA* genetic testing should only be offered after genetic counselling [23]. The AGO-OVAR 16 PGx analysis was planned as an exploratory evaluation, and results were not intended for diagnosis, prevention, or treatment of disease. Consequently genetic counselling was not offered to patients at the time of study entry. It could be argued that genetic counselling could be provided at the point patients are made aware of the availability of their individual *BRCA* results. However, France, Germany, and Scandinavian countries (which between them recruited >40 % of the study patients in AGO-OVAR 16, and >46 % of patients with a *BRCA* mutation were from these countries) indicated that genetic counselling qualifications would be required for investigators to discuss genetic results with their patients, but that not all study investigators had the necessary qualification. There is a recognized shortage of genetic counselors; for instance, there is approximately 1 genetic counselor to every 80,000 people in the United States [30]. Thus, while investigators had resources to contact patients to share results as part of the clinical study, the availability and variability in genetic counselling requirements presented a significant limitation for patients to discuss their research results and seek guidance on follow-up. Ambiguity in the interpretation of individual *BRCA* test results has been reported [31, 32], further emphasizing the need for qualified genetic counselors in communicating genetic research findings such as those described in this study. Alternative genetic counselling models, such as telephone counselling and telegenetics, have been proposed in the oncology setting and may be a suitable approach where resources are limited. However, these approaches still require comparative effectiveness studies to fully evaluate short- and long-term patient outcomes [33] and as such may not have been suitable in this study. Clearly anticipating the need for genetic counselling is a key learning from this study, and in the future, sponsors of clinical trials should carefully consider resource needs and at what stage of the trial they should be incorporated.

### Analytical validity of *BRCA* research results in AGO-OVAR 16

When offering individual test results to research participants, procedures should be in place to ensure that the analyzed sample is actually from the person it is believed to be from [34] and the results should be independently confirmed for accuracy [11]. Indeed, the 2010 Genetic Diagnostic Act of Germany requires that research participants who wish to receive their genetic results must be re-tested at an approved genetic laboratory [35]. Furthermore, in Germany having a research-quality *BRCA* test result would not necessarily qualify a patient for a confirmatory diagnostic test because referral is only available in cases of platinum-sensitive ovarian cancer relapse or where the patient fulfills the eligibility criteria for the consortium of hereditary breast or ovarian cancer. With ever-increasing access to genetic testing through participation in research and the likelihood of incidental findings, it will become necessary to update eligibility criteria where participants obtain research-quality *BRCA* test results. Ensuring that *BRCA* test results are included in eligibility criteria for diagnostic testing may inform a potential preventative approach where disease has not yet developed. In the United States, the Center for Medicare and Medicaid Services (CMS) currently interprets CLIA (Clinical Laboratory Improvement Amendments) regulations as prohibiting the return of non-accredited results for treatment or health-assessment purposes. Furthermore, the return of these results to enable research participants to seek counselling or a confirmatory test in a CLIA-certified laboratory is considered as “treatment” by the CMS. In contrast, for entities covered by the Health Insurance Portability and Accountability Act of 1996 (HIPAA), the recently amended HIPAA “Privacy Rule” provides research participants with broad rights to access their individual results held in designated records irrespective of the environment in which the results were generated, a position that appears to be inconsistent with CLIA-regulation [36]. Thus, with regard to research results that have potential clinical relevance, researchers may be faced with contradiction in law and may be reluctant to return results due to the risks that study participants may misinterpret or misuse them. Such inconsistency clearly requires clarification at both national and international levels as researchers are currently faced with the possibility of violating regulations for whichever approach is taken. Indeed, the US HHS Secretary’s Advisory Committee on Human Research Protections (SACHRP) recently highlighted this inconsistency and recommended urgent clarification to resolve the regulatory conflicts [37], recommendations that still require implementation. Unfortunately at an international level, attempts to harmonize policy and guidelines are overdue and researchers continue to be faced with regional differences.

In the AGO-OVAR 16 study PGx blood samples were collected for exploratory PGx research. PGx blood samples were tracked from the point of collection through DNA extraction and genotyping. Although there was a high degree of confidence that a sample for any given individual was indeed from that individual, the tracking system was not externally validated (but was appropriate for exploratory analyses). *BRCA* genotyping was conducted in non-accredited research laboratories using NGS technologies and reagents designated for research use only. Comparisons with independent array-based genotyping of exonic *BRCA1/2* variants confirmed high accuracy for the NGS approach. Individual genotype data were compared to the BIC database to identify clinically important *BRCA* mutations. While a positive match indicated a high chance that the variant carried by an individual is clinically important, there was a potential for false-negative calls because it is unlikely that the BIC database included all (likely) pathogenic *BRCA* mutations. While samples were still available, they were stored long term in a central repository where sample tracking was not validated for diagnostic purposes; thus, there would be a residual issue of analytical validity if these samples were re-tested in an accredited environment. Essentially, the methods employed were not approved for individual diagnostic purposes but were aligned for population PGx research that sought to understand the relationship between *BRCA* mutation status and response to treatments.

### Returning aggregate *BRCA* research results

**Fig. 1:**
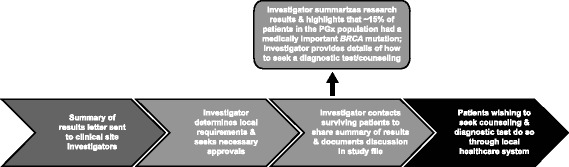
Process for the return of aggregate *BRCA* research results to patients enrolled in AGO-OVAR 16. PGx, pharmacogenetic

Overall, our evaluation recognized the importance of communicating *BRCA* research results to patients; however, many issues (e.g., ICF, local regulation, and guidance) exist that would challenge the appropriateness of proactively communicating individual *BRCA1*/*2* research results to patients in AGO-OVAR 16. Therefore, we considered an alternative approach, advocated by some commentators [38], to proactively communicate the aggregate genetic result to patients via investigators. Patients could then request their individual results if they desired. While aggregate PGx results are frequently reported in peer-reviewed publications, they are rarely provided directly to individual study participants [5, 38]. Although challenges do exist with the communication of aggregate genetic results [5], they are not insurmountable. We developed an approach to return the aggregate *BRCA1*/*2* result to patients via study investigators (Fig. 1). Briefly, the pharmaceutical sponsor provided a “Dear Investigator Letter” (Additional file 3) that summarized the key findings of the PGx research. Based on feedback from local investigators and operating companies, a number of circumstances were identified where it may be inappropriate to contact patients or where local laws restricted the return of research results. Thus, investigators were not mandated to share the aggregate *BRCA1/2* study findings, and a mechanism for tracking what the investigators did was therefore not established. Study investigators were encouraged to contact surviving study patients to inform them of the overall *BRCA1*/*2* findings in relation to PFS, and, based on local guidelines, test availability and individual circumstances, to discuss whether it would be appropriate to seek genetic counselling and a diagnostic quality *BRCA1*/*2* test. Study patients would benefit from understanding the research, why it was conducted, the implications to them and their family, and would be in a better position to make a more informed decision regarding follow-up. Contact with family members of deceased trial participants was not recommended because it was outside the scope of the informed consent and thus, by law, not allowed in some countries or allowed only in exceptional circumstances in other countries [39]. Additional circumstances under which it may be inappropriate to contact participants included patient withdrawal of consent, previous *BRCA* testing by a diagnostic laboratory, or progressive disease such that *BRCA* status would not change or affect decision on further treatment options. Investigators were asked to determine local requirements and seek necessary approvals prior to making contact with surviving study participants.

## Discussion

Technological advances provide an opportunity to gain greater understanding of the relationship between genetic variation, disease, and response to medicines. However, the increasingly routine approaches of whole-exome or whole-genome sequencing using NGS, where the analysis aims to identify rare variants associated with drug response, heightens the probability of incidental findings and the discovery of variants of unknown significance [31]. This potentially increases the burden on local investigators and counselling and testing facilities and raises questions of how researchers should manage genetic results. The ongoing ethical debate regarding the benefits, risks and utility of returning individual genetic research-quality results has generally failed to consider the complexities of working in an international clinical trial setting, where variations exist in regulation, guidance, and ethical opinion. Guidance for the communication of results exists, but there is a lack of agreement and a need for harmonization across countries [9].

At the time the AGO-OVAR 16 study was set up, general practice was not to communicate individual exploratory PGx results. This approach was aligned with ethical guidance in many countries on the handling of exploratory genetic data [5] and with EC opinion, which often required consent forms to stipulate that individual exploratory genetic results would not be returned [10]. In AGO-OVAR 16, scientists, investigators, and representative physicians involved in the care of patients were directly involved in the decision process, and a wide variety of diverse perspectives were represented. These opinions varied from those advocating the complete proactive communication of individual *BRCA* research results to all patients to those advocating no proactive communication at all. Very real challenges were encountered in reaching a consensus opinion that addressed all viewpoints yet was consistent with constraints from the historic trial design. While the communication of individual *BRCA1*/*2* data may provide benefit for some study patients, and was considered, the fact that the initial consenting process did not involve a discussion about the return of research-quality PGx results was viewed as a significant barrier to communicating individual results. Furthermore, a clinical decision to conduct diagnostic testing for *BRCA* mutation status would be influenced by specific local eligibility criteria at the time of diagnosis. Patients at highest risk of carrying a mutation in *BRCA1/2* (e.g., those with a family history and/or early age of disease onset) are more likely to meet the eligibility criteria for a diagnostic test, and thus many patients identified in this study as *BRCA-*positive may already be aware of their *BRCA* status. It is therefore not clear that access to a research test result several years after initial diagnosis would alter this initial decision or meet eligibility criteria for diagnostic testing in some countries. Our assessment highlighted that while there may be good cause to return individual *BRCA* research test results, particularly for patients called as *BRCA-*positive, we were nonetheless constrained by local requirements restricting the return of research results. Importantly, the informed consent document presented patients an opportunity to request individual results generated during the study. Thus any patient would be able to request their individual *BRCA* research test result after receiving the aggregated result. It could be argued that our approach to proactively communicate the aggregate research result rather than proactively communicate individual results to study participants may not be optimal with regard to researcher’s ethical duty to patients as it relies on patients taking the initiative to request their own data. However, in our view, the pragmatic approach taken here provided patients with the opportunity to request their individual data, thus fulfilling the obligation of autonomy and complying with local restrictions and the ICF.

An acknowledged limitation of our approach is that patient advocacy groups were not involved in the decision process. In hindsight, such input could have provided valuable insights to inform our understanding of international variability in patients’ attitudes towards the return of individual genetic research results and may have served to heighten debate on this difficult topic. It is, however, unclear how this would have modified the ultimate decision taken for AGO-OVAR 16 given the constraints identified. We hope that the issues highlighted in this paper will help to stimulate discussions involving cross-disciplinary parties, including patient advocacy groups.

The return of aggregate results provides a mechanism to convey research findings to patients, which demonstrates reciprocity where the sharing of individual results is not appropriate [38] and fulfills ethical obligations found in the Helsinki declaration [9]. Although it is not yet clear whether exploratory PGx research results are in scope, Clinical Trial Regulation (CTR) EU No. 536/2014 [40] will require sponsors to provide a lay summary of aggregate clinical trial results for submissions in Europe, further emphasizing an increased desire for greater transparency. Study investigators have the benefit of a direct relationship with the patient, thereby understanding their individual circumstances (e.g., whether they have previous diagnostic-quality *BRCA1*/*2* test result), and are best suited to describe the aggregate findings in appropriate lay terms and to highlight key distinctions between research and healthcare settings, thus helping to avoid potential therapeutic misconception that could result in harm, including emotional distress. Furthermore, they have an understanding of clinical practice in terms of local counselling and confirmatory testing options. Feedback from one country highlighted that investigators communicated the aggregate results to all patients together with the recommendation to seek further explanation from a genetic counselor. More than 50 % of patients in this country did seek follow-up but encountered limited availability of local genetic counselling and testing facilities. This experience highlights some of the constraints that investigators and patients may encounter when seeking specialist resources and emphasizes the need to consider these challenges during the design of new clinical trials, particularly in an international setting where access to resources may vary between countries.

The sharing of aggregate results may trigger a desire from participants to know their individual *BRCA* result if they do not already have a diagnostic-quality test result. Thus, by sharing aggregate results it will be necessary to prepare for requests for individual *BRCA* results. Requests would be handled through the patient’s study investigator, and individual *BRCA* results would carry the caveat that they were not intended for the diagnosis, prevention, or treatment of disease.

Study participants who request and obtain their *BRCA1*/*2* research results (or who have a diagnostic quality *BRCA1*/*2* test result) may choose to subsequently share the information with relatives. The potential for relatives to make an informed decision to seek their own diagnostic quality *BRCA1*/*2* test and subsequent follow-up (e.g., seek risk-reducing surgery) may arguably be considered a significant benefit of returning *BRCA1*/*2* information to patients who already have ovarian cancer. However, the relationship between clinical study patients and researchers often does not extend to family members, and interaction between researchers and family members was outside the scope of the AGO-OVAR 16 consent. Furthermore, the sharing of research results directly with relatives (e.g., where the patient is deceased) would be contrary to law in many countries were legislation exists [39]. Although the approach taken here may be considered controversial, there is nonetheless a clear challenge for researchers who are faced with the dilemma of conflicting ethical and legal frameworks. The occurrence of unexpected incidental findings is hardly new and is likely to recur in future studies. We would argue that current law and guidelines should be revised to provide researchers with a clear picture of how to proceed where there is a strong ethical argument to share results with patients or their relatives but where the sharing of such data was unanticipated.

Although resources to communicate the aggregate result to patients were in place, these did not extend to genetic counselling or confirmatory diagnostic-quality testing, which was outside the scope of the study protocol. Future clinical trial protocols should describe clearly the possibility that genetic research data may be communicated under some circumstances and describe the mechanism of dissemination. The ICF should provide research participants with the opportunity to “opt-in” or “opt-out” with respect to the communication of individual genetic research results. This approach would need to take into account different requirements and resources for genetic counselling and confirmatory testing between jurisdictions and consider what action to take where a participant initially opted to receive medically important research findings, but later withdrew from the study or became lost to follow-up. Our experience demonstrates that a single approach for all participants enrolled in international studies may be difficult to achieve, and that it may be necessary for local solutions to be developed based on locally available resources, requirements, and ethical guidance.

Ideally individual genetic results should be returned when the clinical trial produces clinically useful data for a validated biomarker [11] and when the research result is analytically valid [41]. However for clinical trials, changes to current processes would be required to ensure study participants understand the circumstances under which their genetic research results would be returned. In future clinical trials, during study enrolment, sponsors would specify that medically important genetic research results will be returned to participants who wish to receive them. Study sponsors could then take one of a number of approaches (A, B, and C) as summarized in Table 2.

**Table 2:** Approaches to disseminate medically important genetic research results

Option A: In the event of a medically important finding when conducting exploratory genetic research, contact participants and seek consent to collect a second sample for the purpose of a diagnostic confirmatory test.	
Option B: Conduct all aspects of genetic research in an accredited environment.	
Option C: Communicate medically relevant genetic research results to study participants who opt to receive their individual genetic research results, via clinical investigator and after appropriate genetic counselling. Advise study participants that they would need to seek a diagnostic confirmatory test.	

While approach (A) may be desirable, the burden on sponsors, clinical site staff, and study participants to reconnect, re-sample, and re-test in an accredited environment, perhaps many years after participants ended their involvement in the study, presents practical and resource challenges. Approach (B) may negate the need to reconnect with study participants but would increase study costs and resources. Because the primary purpose of genetic research is not diagnostic in nature, recommendations requiring all clinical trials to be set up to enable the independent validation of individual genetic data or to conduct research in an accredited environment (when in the vast majority of cases the research is exploratory), are unlikely to be adopted voluntarily. Alternatives to collecting and storing participant samples in an accredited environment and taking an aliquot for exploratory genetic research could be considered, but again such alternatives would incur additional study costs when for the majority of cases, a confirmatory test would not be required. Where a clinical trial incorporates a predefined genetic research objective that involves testing of genetic markers with an established clinical utility, there is a strong argument that the genetic research should be conducted in an accredited environment that meets recognized criteria for analytical validity.

As long as it is clear to study participants who opt to receive their individual genetic research results that the responsibility for seeking a confirmatory diagnostic test does not reside with researchers, we would advocate approach (C). The responsibility for interpreting individual results would reside with the participant in consultation with qualified specialists who can evaluate the genetic research results and future risk for conditions that are likely multi-factorial. Furthermore, this approach will enable study participants to seek the follow-up most appropriate to their personal circumstances and local environment. While fulfilling a responsibility of researchers and physicians to share clinically relevant research results, this approach would minimize the need to maintain study resources, potentially for many years after conclusion of the clinical study, and is likely to be more desirable for sponsors and clinical site investigators. This approach also offers the opportunity for investigators to track and maintain contact with research participants who opt to be informed of incidental findings that may affect their health or that of their relatives. Indeed, if the informed consent process had provided patients with the option to be re-contacted to provide their provisional *BRCA* research test result, a different approach may have been possible in AGO-OVAR 16. It may have presented an opportunity to share individual results specifically for those patients called as carrying a medically important *BRCA* mutation and who opted to be contacted in these circumstances. Irrespective of the approach taken, sponsors would need to remain cognizant of regulations and guidance that restrict return of research results (e.g., CLIA-regulation in the US) and that differences in requirements may exist between countries. It will be necessary to tailor the approach based on local law, regulation, and EC advice.

## Conclusion

Our experiences highlight the need for careful consideration and awareness of local law, clinical practice, and ethics guidance, all of which may differ between countries. In international clinical trial settings, a single approach may not be appropriate when considering communication of individual genetic research results. At the time the AGO-OVAR 16 protocol was developed, the ethical, regulatory, and professional consensus was to not return genetic research results to study participants. This position has subsequently evolved and will continue to do so in the future. Using our experience from AGO-OVAR 16, a process was developed for the return of aggregate PGx research results to clinical trial participants that can be used in future clinical trials. While the approach taken in this study is not without its limitations, and opinions on the merits of the approach will vary, it was considered a reasonable approach given the lack of consensus of opinion. This study does highlight the need for further debate and the need to resolve current legal inconsistencies at national and international levels. Our experience highlights that post-hoc decisions regarding the communication of genetic research results are seldom satisfactory. Given the rapid pace of change in genetic research it is likely scenarios will continue to arise that were unanticipated at study design. A recent proposal to establish a secondary-genomic-findings service to support researchers in the return of individual clinically actionable genetic research findings may provide one solution in these cases [2]. Our study illustrates the importance of increasing the clarity and scope of informed consent to ensure clinical trial participants can indicate their preference regarding receipt of potentially important individual genetic results and to account for unanticipated “incidental findings.” Future clinical trials should proactively consider the strategy for communicating genetic research data to patients and the environment (accredited or non-accredited) in which genetic research is to be conducted.

## Additional files

Additional file 1: Table S1. Lists names and affiliations of local Institutional Review Boards and Independent Ethics Committees that approved the study. (PDF 211 kb)
Additional file 2: Table S2. Shows the countries and respective populations that were involved in AGO-OVAR 16. (DOC 28 kb)
Additional file 3: Letter from GSK and the Trial Steering Committee to investigators who participated in AGO-OVAR 16. The letter outlines actions to be taken by GSK and those that are required by the investigators. (PDF 956 kb)

## Abbreviations

BIC: Breast Cancer Information Core
EC: Ethics committee
ICF: Informed consent form
NGS: Next-generation sequencing
PARP: Poly ADP ribose polymerase
PFS: Progression-free survival
PGx: Pharmacogenetic
SNV: Single nucleotide variant
