# Supplementary material for: Communicating BRCA research results to patients enrolled in international clinical trials: lessons learnt from the AGO-OVAR 16 study
Source: BMC Med Ethics. 2016 Oct 21;17:63. doi: 10.1186/s12910-016-0144-y (PMC5073453; doi:10.1186/s12910-016-0144-y)
Supplement: Additional file 1: Table S1. — Lists names and affiliations of local Institutional Review Boards and Independent Ethics Committees that approved the study. (PDF 211 kb) [file 12910_2016_144_MOESM1_ESM.pdf]

Table S1: Names and affiliations of local institutional review boards and ethics committees that approved AGO-OVAR 16.

Country	Institutional review board/ethics committee name and affiliation
Australia	Bellberry Human Research Ethics Committee, 229 Greenhill Road, Dulwich South Australia, 5065, Australia
	Cabrini Institute Human Research Ethics Commitee, 183 Wattletree Road, Malvern, Victoria, 3144, Australia
	Cancer Institute New South Wales Clinical Research Ethics Committee, Australian Technology Park, Biomedical Building, Suite 101, 1 Central Avenue (off Garden Road), Eveleigh, New South Wales, 2015, Australia
	Human Research Ethics Office, Level 7, Block 7, Royal Brisbane and Women's Hospital, Australia
	Mercy Health Human Research Ethics Committee, Mercy Hospital for Women, 163 Studley Road, Heidelberg, 3084, Australia
	Royal Adelaide Hospital Research Ethics Commitee, Level 3, Hanson Institute, North Terrace, Adelaide, South Australia, 5000, Australia
	Royal Women's Hospital Human Research Ethics Committee, The Royal Women's Hospital, Corner Grattan Street & Flemington Road, Parkville, Victoria, 3052, Australia
	Sir Charles Gairdner Group Human Research Ethics Committee, Sir Charles Gairdner Hospital, Hospital Avenue, Nedlands, Western Australia, 6009, Australia
	Tasmania Health and Medical Human Research Ethics Committee, Research House, Sandy Bay Campus, University of Tasmania, Tasmania, Australia
Austria	Ethikkommission der Med. Universität Innsbruck, Geschäftsstelle, Innrain 43, Innsbruck, A-6020, Austria
Belgium	Commissie Medische Ethiek-toetsingcommissie, UZ Leuven, Campus Gasthuisberg E330, Herestraat 49, Leuven, 3000, Belgium
China	Ethics Commettee of Beijing 301 PLA Hospital, NO.28, Fu Xing Avenue, Haidian District, Beijing, 100853, China
	Ethics Commettee of Cancer Hospital of Jiangsu Province, No.42 Baiziting, Nanjing, Jiangsu, 210009, China
	Ethics Commettee of Cancer Hospital, CAMS and PUMC, No.17, Pan Jia Yuan Nan Li, Chaoyang District, Beijing, 100021, China
	Ethics Commettee of Obstetrics and Gynecology Hospital Of Fudan University, Room 308 3F No.506 Fangxie Road, Huangpu District, Shanghai City, 200011, China
	Ethics Commettee of Peking University People's Hospital, Peking University People's Hospital, No.11, Xizhimen South Avenue, Xicheng District, Beijing, 100044, China
	Ethics Commettee of Shengjing Hospital of China Medical University, No.36, Sanhao Street, Heping District, Shenyang City, Liaoning Province, 110004, China
	Ethics Commettee of Tianjin Cancer Hospital, 2/F, Cancer Institute, No.47, Binshui Dao, Tiyuan Bei, Hexi District, Tianjin, 300060, China
	Ethics Committee of Beijing Cancer Hospital, No.52, Fu Cheng Road, Haidian District, Beijing, 100141, China
	Ethics Committee of Cancer Hospital Affiliated to Fudan University, No.270, Dong An Road, Shanghai, 200032, China
	Ethics Committee of Women's Hospital of Zhejiang Unviersity, No.1, Xueshi Road, Hangzhou, Zhejiang Province, 310006, China
	Ethics Committee of Zhejiang Cancer Hospital, No.38, Guangji Road, Banshan Bridge, Hangzhou, 310022, China
	Ethics Committee of Sun Yat-Sen University Cancer Center, 651, Dongfeng East Road, Guangzhou, 510060, China
	Shandong University Qi Lu Hospital, No.107, Wenhua West Road, Li Xia District, Jinan, Shandong, 250012, China
Denmark	De Videnskabsetiske Komiteer i Region Hovedstaden, Kongens Vaenge 2, Hilleroed, 3400, Denmark
France	CPP Sud-Ouest et Outre Mer III, Service de Pharmacologie Clinique, Bat. 1A, Hopital Pellegrin, Place Amelie Raba Leon, Bordeaux Cedex, 33076, France
Germany	Ethikkomission der Landesaerztekammer Hessen, Im Vogelgesang 3, Frankfurt, 60488, Germany
Hong Kong	Institutional Review Board of the University of Hong Kong/Hospital Authority Hong Kong West Cluster, Queen Mary Hospital, Room 901, Administration Block, 102 Pokfulam Road, Hong Kong, Hong Kong
	Research Ethics Committee(Kowloon Central/Kowloon East), Queen Elizabeth Hospital, 30 Gascoigne Road, Kowloon, Hong Kong
Ireland	Clinical Research Ethics Committee of the Cork Teaching Hospital, Lancaster Hall, 6 Little Hanover Street, Cork, Ireland
	Comitato di Bioetico, Ospedale S. Giovanni Calibita Fatebenefratelli di Roma, Isola Tiberina, 39, Roma, Lazio, 00186, Italy
	Comitato Etico Azienda Spedali Civili di Brescia, Piazzale Spedali Civili, 1, Brescia, Lombardia, 25123, Italy
	Comitato Etico Centro di Riferimento Oncologico, Istituto Nazionale Tumori (IRCCS) di Aviano, Segreteria Scientifico-Amministrativa, Via Pedemontana Occidentale, 12, Aviano (PN), Friuli-Venezia-Giulia, 33081, Italy
	Comitato Etico dell' Azienda Ospedaliera della Valtellina e della Valchiavenna, Via Stelvio, 25, Sondrio, Lombardia, 23100, Italy
	Comitato Etico dell' Azienda Ospedaliera San Carlo, Via Potito Petrone, Potenza, Basilicata, 85100, Italy
	Comitato Etico dell' Indipendente Locale, Azienda Ospedaliera Universitaria Consorziale, Segreteria Scientifico-Amministrativa, Piazza Giulio Cesare, 11, Bari, Puglia, 70124, Italy
	Comitato Etico dell' Istituto di Ricovero e Cura a Carattere Scientifico, Ospedale Maggiore Policlinco Mangiagalli e Regina Elena, Via Francesco Sforza, 28, Milano, Lombardia, 20122, Italy
	Comitato Etico dell’ Azienda Ospedaliera Ospedali Riuniti Villa Sofia-Cervello, Via Trabucco 180, Palermo, Sicilia, 90146, Italy
	Comitato Etico dell’Azienda Ospedaliera, San G. Moscati, Contrada Amoretta, Città Ospedaliera, Pal. Uffici, Avellino, Campania, 83100, Italy
	Comitato Etico della AUSL di Bologna, c/o Dipartimento Farmaceutico, Via Gramsci, 12, Bologna, Emilia-Romagna, 40121, Italy
	Comitato Etico della Fondazione Centro S. Raffaele del Monte Tabor (Istituto di Ricovero e Cura a Carattere Scientifico) di Milano, Dipartimento Medicina Interna, Via Olgettina 60, Milano, Lombardia, 20132, Italy
	Comitato Etico Della Provincia Di Modena, Via Largo del Pozzo 71, Modena, 411204, Italy
	Comitato Etico dell'Azienda Ospedaliera San Gerardo di Monza, Via Pergolesi, 33, Monza, Lombardia, 20052, Italy
	Comitato Etico dell'Azienda Ospedaliera Sant'Anna di Como, Segreteria Scientifico-Amministrativa, Via Napoleona, 60, Como, Lombardia, 22100, Italy

Italy	Comitato Etico delle Azinede Sanitarie dell'Umbria di Perugia, via della Rivoluzlone,l, ellera di Corciano, Perugla, 06070, Italy
	Comitato Etico dell'lstituto Europeo di Oncologia, Via Ripamonti 435, Milano, 20141, Italy
	Comitato Etico dell'Universita' Cattolica del Sacro Cuore -Policlinico Gemelli, Largo Agostino Gemelli, n.8, Roma, Lazio, 00168, Italy
	Comitato Etico di Area Vasta Romagna di Cesena, Piazza Leonardo Sciascia, 111/2, Cesena (FC), Emilia-Romagna, 47023, Italy
	Comitato Etico I.FO.Instituto Fisioterapici Ospitalieri Istituto di Ricovero e Cura a Carattere Scientifico Regina Elena, Istituto di Ricovero e Cura a Carattere Scientifico San Gallicano, Via E.Chianesi 53, Roma, Lazio, 00144, Italy
	Comitato Etico Indipendente dell' Azienda Ospedaliero-Universitaria di Bologna, Policlinico Sant'Orsola Malpighi, Via Albertoni 15, Padiglione 3, Bologna, Emilia-Romagna, 40138, Italy
	Comitato Etico Indipendente Istituto Nazionale per lo Studio e la Cura dei Tumori 'Fondazione Giovanni Pascale' (IRCCS), Via Mariano Semmola, 3, Napoli, Campania, 80131, Italy
	Comitato Etico Indipendente, Fondazione Istituto di Ricovero e Cura a Carattere Scientifico Istituto dei Tumori, Segreteria Scientifico-Amministrativa, Via Venezian, 1, Milano, Lombardia, 20133, Italy
	Comitato Etico Interaziendale delle ASO Ospedale Infantile Regina Margherita Sant Anna e Ordine Mauriziano di Torino, Corso Spezia, 60, Torino, Piemonte, 10126, Italy
	Comitato Etico Istituto Europeo di Oncologia, Via Ripamonti, 435, Milano, Lombardia, 20141, Italy
	Comitato Etico Ospedale di Circolo, Viale Borri, Varese, 57-21100, Italy
	Comitato Etico per la Sperimentazione Clinica della Provincia di Vicenza, Ospedale San Bortolo, Via Rodolfi, 37, Vicenza, Veneto, 36100, Italy
	Comitato Etico per le Attività Biomediche -Università degli Studi di Napoli Federico II, Segreteria Scientifico-Amministrativa, Via Pansini, 5, Napoli, Campania, 80131, Italy
	Comitato Etico Provinciale di Reggio Emilia, Segreteria Scientifico-Amministrativa, Viale Risorgimento, 57, Reggio Emilia, Emilia-Romagna, 42100, Italy
	Commissione Etico-Scientifica dell'Ospedale Niguarda Cà Granda, Segreteria Scientifico-Amministrativa, Piazza Ospedale Maggiore, 3, Milano, Lombardia, 20162, Italy
	Etico dell'Universita' Cattolica del S. Cuore -Policlinico Gemelli, Largo Gemelli, 8, Roma, Lazio, 168, Italy
Japan	Institutional Review Board, Hiroshima University Hospital, 1-2-3, Kasumi, Minami-ku, Hiroshima-city, Hiroshima, 734-8551, Japan
	Institutional Review Board, Hokkaido University Hospital, North 14, West 5, Kita-ku, Sapporo-shi, Hokkaido, 060-8648, Japan
	Institutional Review Board, Kagoshima City Hospital, 20-17, Kajiya-cho, Kagoshima-shi, Kagoshima, 892-8580, Japan
	Institutional Review Board, Keio University Hospital, 35, Shinanomachi, Shinjuku-ku, Tokyo, 160-8582, Japan
	Institutional Review Board, Kinki University Hospital, 377-2, Ohnohigashi, Osakasayama-city, Osaka, 589-8511, Japan
	Institutional Review Board, National Cancer Center Hospital, 5-1-1, Tsukiji, Chuo-ku, Tokyo, 104-0045, Japan
	Institutional Review Board, National Hospital Organization Kyushu Cancer Center, 3-1-1, Notame, Minami-ku, Fukuoka-city, Fukuoka, 811-1395, Japan
	Institutional Review Board, National Hospital Organization, Shikoku Cancer Center, 160, Kou, Minamiumemotomachi, Matsuyama-shi, Ehime, 791-0280, Japan
	Institutional Review Board, Saitama Medical University International Medical Center, 1397-1, Yamane, Hidaka-city, Saitama, 350-1298, Japan
	Institutional Review Board, The Jikei University School of Medicine, 3-19-18, Nishishinbashi, Minato-ku, Tokyo, 105-8471, Japan
	Institutional Review Board, Tohoku University Hospital, 1-1, Seiryo-machi, Aoba-ku, Sendai-city, Miyagi, 980-8574, Japan
	Institutional Review Board, Tottori University Hospital, 36-1, Nishi-cho, Yonago-City, Tottori, 683-8504, Japan
	Iwate Medical University Hospital Institutional Review Board, 19-1, Uchimaru, Morioka-city, Iwate, 020-8505, Japan
	National Hospital Organization Kure Medical Center and Chugoku Cancer Center Institutional Review Board, 3-1, Aoyama-cho, Kure-city, Hiroshima, 737-0023, Japan
Norway	Regional komite for medisinsk og helsefaglig forskningsetikk, Sor-Ost-Norge (REK Sor-Ost), Pb. 1130 Blindern, Oslo, 03180,Norway
Republic of Korea	Asan Medical Center Institutional Review Board, 388-1 Pungnap-2-Dong, Songpa-Gu, Seoul, 138-736, Korea
	Institutional Review Board of Samsung Medical Center, 50 Ilwon-Dong, Kangnam-Ku, Seoul, 135-710, Korea
	Institutional Review Board of Seoul National University Hospital, 28 Yungun-Dong Chongno-Ku, Seoul, 110-744, Korea
	National Cancer Center Institutional Review Board, 809, Madu-dong, Ilsan-gu, Goyang-si, Gyeonggi-do, 410-769, Korea
	Institutional Review Board of Kangnam Severance Hospital, 146-92, Dogok-Dong, Kangnam-Ku, Seoul, 135-702, Korea
	Comite Etico de Investigacion Clinica (CEIC), Grupo Hospitales de Madrid, Avenida Castillo Olivares, sIn, Torrelodones, Madrid, 28250, Spain
	Comite Etico de Investigacion Clinica de Euskadi, Direccion de Farmacia, Departamento de Sanidad del Gobierno Vasco, C/ Donostia-San Sebastian, n0 1, Vitoria , 01010, Spain
	Comite Etico de Investigacion Clinica, Arnau de Vilanova, Avenida Alcalde Rovira Roure, 80, Lérida, 25198, Spain
	Comite Etico de Investigacion Clinica, Clinico de Santiago, Choupana s/n, Santiago de Compostela, 15706, Spain
	Comite Etico de Investigacion Clinica, Corporació Sanitària Parc Taulí, C/Parc Taulí, s/n, Sabadell, 08208, Spain
	Comite Etico de Investigacion Clinica, Fundación Hospital Alcorcón, Budapest, 1, Alcorcón (Madrid), 28922, Spain
	Comite Etico de Investigacion Clinica, Hospital de Donosti, Paseo Dr. Beguiristain, 109, San Sebastián, 20014, Spain
	Comite Etico de Investigacion Clinica, Hospital de Navarra, Irunlarrea, 3, Pamplona, 31008, Spain
	Comite Etico de Investigacion Clinica, Hospital de Terrassa, Carretera de Torrebonica s/n, Terrassa, Barcelona, 08227, Spain
	Comite Etico de Investigacion Clinica, Hospital del Mar, Paseo Marítimo, 25-29, Barcelona, 08003, Spain
	Comite Etico de Investigacion Clinica, Hospital General de Valencia, Avenida Tres Cruces s/nº, Valencia, 46014, Spain

Spain	Comite Etico de Investigacion Clinica, Hospital Gregorio Marañón, C/ Dr. Esquerdo, 46, Madrid, 28007, Spain
	Comite Etico de Investigacion Clinica, Hospital La Paz, Paseo de la Castellana, 261, Madrid, 28046, Spain
	Comite Etico de Investigacion Clinica, Hospital Miguel Servet, Pº Isabel La Catolica 3, Zaragoza, 50009, Spain
	Comite Etico de Investigacion Clinica, Hospital Ramon y Cajal, Ctra. Colmenar Viejo Km 9.1, Madrid, 28034, Spain
	Comite Etico de Investigacion Clinica, Hospital Santa Creu i Sant Pau, Servicio de Hematologia, Avda. Sant Antoni Maria Claret, 167, Barcelona, 8025, Spain
	Comite Etico de Investigacion Clinica, Hospital Son Llatzer, Ctra. de Manacor, 4, Palma de Mallorca, 07198, Spain
	Comite Etico de Investigacion Clinica, Hospital Universitario La Fe, Bulevar sur s/n, Valencia, 46026, Spain
	Comite Etico de Investigacion Clinica, Hospital Vall d' Hebrón, Edificio Instituto Recerca 2ª planta, Paseo de la Vall d'Hebrón, 119-129, Barcelona, 08035, Spain
	Comite Etico de Investigacion Clinica, Hospital Virgen de la Arrixaca, Ctra. Madrid-Cartagena, s/n, Murcia (El Palmar), 30120, Spain
	Comite Etico de Investigacion Clinica, Hospital Virgen del Rosell, Paseo Alfonso XIII, 61, Cartagena , Murcia, 30203, Spain
	Comite Etico de Investigacion Clinica, Instituto Valenciano de Oncologia, C/ Profesor Beltrán Báguena 11, 8 y 19, Valencia, 46009, Spain
	Comite Etico de Investigacion Clinica, Ruber Internacional, C/La Maso 38, Madrid, 28034, Spain
	Comite Etico de Investigacion Clinica, Ciutat Sanitaria y Universitaria de Bellvitge, Edificio de Consultas Externa, 1ª planta, C/Feixa, Llarga, s/n, L´Hospitalet de Llobregat, Barcelona, 08907, Spain
	Hospital General de Elche, C/ Camí de la Almazara, Elche, 03203, Spain
Sweden	Regionala Etikprövningsnämnden i Uppsala, Drottninggatan 4, SE-753 09 Uppsala, Sweden
Taiwan	Institution of Review Board, Mackay Memorial Hospital, No.92, Section 2, Chung-Shan North Road, Taipei, 104, Taiwan
	Institution of Review Board, Taipei Veterans General Hospital, , No.201, Section 2, Shih-Pai Road, Taipei, 112, Taiwan
USA	Atlantic Health System Inc., Institutional Review Board, 475 South Street, Morristown, New Jersey, 07962, United States
	City of Hope Institutional Review Board, 1500 East Duarte Road, Duarte, California, 91010-3000, United States
	Columbia University Medical Center Institutional Review Board, 722 West 168th Street, MSPH room 426, New York, 10032, United States
	Committee on Human Research, University of California, San Francisco, Box 0962, 3333 California Street, Suite 315, San Francisco, California, 94143-0962, United States
	Human Subjects Review Committee, University of California, Davis CTSC Building, 2921 Stocklon Boulevard, Suite 1400, Sacramento, California, 95817, United States
	Institutional Review Board at Montefiore, Office of Research and Sponsored Programs, 3308 Rochambeau Avenue, Bronx, New York, 10461, United States
	Institutional Review Board, Kaiser Permanente Southern California, 393 East Walnut Street, 2nd Floor, Pasadena, California, 91188, United States
	Kaiser Foundation Research Institute, Kaiser Permanente Northern California Institutional Review Board, 1800 Harrison Street, Oakland, 16th Floor, California, 94612-3433, United States
	Long Beach Memorial Medical Center, Memorial Health Service Research Council, 2801 Atlantic Avenue, Long Beach, California, 90801, United States
	Office of Research Integrity and Assurance, 407 East, 61st Street, 1st Floor, New York, 10065, United States
	Office of Research, 5171 California Avenue, Suite 150, Irvine, California, 92697-7600, United States
	Office of the Human Research Protection Program, University of California-Los Angeles, 11000 Kinross Avenue, Suite 102, Box 951694, Los Angeles, California, 90095-1694, United States
	Georgia Regents University Institutional Review Board, Georgia health Sciences University, Human Assurance Committee, 1120 15th Street, CJ-2103, Augusta, Georgia, 30912-7621, United States
	The University of Texas M.D. Anderson Cancer Center Surveillance Committee -FWA 363, 1515 Holcombe Boulevard, Unit 198, Houston, Texas, 77030-4009, United States
	Western Institutional Review Board, 3535 Seventh Avenue Southwest, Olympia, Washington, 98502, United States
