# Supplementary material for: Communicating BRCA research results to patients enrolled in international clinical trials: lessons learnt from the AGO-OVAR 16 study
Source: BMC Med Ethics. 2016 Oct 21;17:63. doi: 10.1186/s12910-016-0144-y (PMC5073453; doi:10.1186/s12910-016-0144-y)
Supplement: Additional file 2: Table S2. — Shows the countries and respective populations that were involved in AGO-OVAR 16. (DOC 28 kb) [file 12910_2016_144_MOESM2_ESM.doc]

**Supplementary Information**

**Table S2.** Countries involved in AGO-OVAR 16 contributing to pharmacogenetic population

| **Region** | **Countries** | **Total number of pharmacogenetic samples available for *BRCA* genotyping by region** |
| --- | --- | --- |
| Asia | Australia, China, Hong Kong, Japan, Rep. of Korea, Taiwan | 209 |
| Europe | Austria, Belgium, Denmark, France, Germany, Ireland, Italy, Norway, Spain, Sweden | 431 |
| North America | United States | 24 |
