# Supplementary material for: Communicating BRCA research results to patients enrolled in international clinical trials: lessons learnt from the AGO-OVAR 16 study
Source: BMC Med Ethics. 2016 Oct 21;17:63. doi: 10.1186/s12910-016-0144-y (PMC5073453; doi:10.1186/s12910-016-0144-y)
Supplement: Additional file 3: — Letter from GSK and the Trial Steering Committee to investigators who participated in AGO-OVAR 16. The letter outlines actions to be taken by GSK and those that are required by the investigators. (PDF 956 kb) [file 12910_2016_144_MOESM3_ESM.pdf]

GLAXOSMITHKLINE ADVISORY

Date: 22 April 2015

Dear Investigator,

Title: Germline *BRCA1/BRCA2* Mutation Analysis and Results for Subjects in VEG110655 (OVAR-16) and VEG114012 [A Phase III Study to Evaluate the Efficacy and Safety of Pazopanib Monotherapy Versus Placebo in Women Who Have not Progressed after First Line Chemotherapy for Epithelial Ovarian, Fallopian Tube, or Primary Peritoneal Cancer, and the Asian Sub-Study]

Therapeutic Indication/Area of Investigation – Ovarian Cancer

Key Messages

- GSK and the Trial Steering Committee is informing all investigators who participated in OVAR16 (VEG110655) and/or the Asian sub-study (VEG114012) that pharmacogenetic research results for the effect of clinically important mutations in the *BRCA1* and *BRCA 2* genes have been recently presented at 15th Biennial Meeting of the Gynecologic Cancer Society, November 8-11, 2014.
- In subjects who consented to and provided blood samples for pharmacogenetic analysis, approximately 15% of subjects were identified as having a clinically important mutation in the *BRCA1* or *BRCA2* gene.
- Results from this pharmacogenetic analysis indicated that clinically important mutations in either *BRCA1* or *BRCA2* is a strong predictor for progression-free survival among subjects enrolled in these studies and had pharmacogenetic data.
- The method used in this pharmacogenetic analysis to determine *BRCA1* and *BRCA2* mutation status is for research purposes only, and was not conducted in a laboratory certified to perform genetic mutation testing. Therefore, these results cannot be considered definitive for any individual patient, and should be confirmed in an accredited laboratory for definitive *BRCA* gene mutation testing.
- Given these results, GSK and the Trial Steering Committee are recommending that investigators contact all subjects they enrolled in this study to consider *BRCA1* and *BRCA2* testing to determine their mutation status.
- Subjects who have withdrawn their consent from further participation in the main clinical studies should not be contacted. Families of deceased subjects should not be contacted because disclosure of trial results as outlined in the Informed Consent Form does not extend to family members to discuss study results.

Action Being Taken by GlaxoSmithKline

GSK and the OVAR-16 Trial Steering Committee (TSC) are contacting all investigators who enrolled subjects in the main study and/or the Asian sub-study. GSK and the TSC agree that investigators should consider contacting their surviving patients and inform them that approximately 15% of subjects in the study were found to have a clinically important mutation in either the *BRCA1* or *BRCA2* gene. Given the importance of these results on the prognosis of a patient in the study, GSK and the TSC are encouraging subjects to have a diagnostic test done for *BRCA1* and *BRCA2* mutations after they have had appropriate genetic counseling where it is available and according to local practices.

Action required by Investigators

Investigators who enrolled subjects in OVAR-16 (VEG110655) and/or the Asian sub-study (VEG114012) should consider contacting surviving patients they enrolled in this study and inform them of these results of the study:

1. Approximately 15% of subjects in these two studies who provided PGx samples were found to have a clinically important mutation in either the *BRCA1* or *BRCA2* genes.
2. The presence of a clinically important mutation in *BRCA1* or *BRCA2* gene was associated with longer progression-free survival for patients enrolled in these two studies and had pharmacogenetic data.

Investigators are encouraged to discuss with their patients that if the patient has not had prior genetic testing, that they consider undergoing genetic counseling and a *BRCA* test in a diagnostic laboratory where it is available and according to local practices.

There may be additional circumstances in which an investigator may feel it is inappropriate to contact their patient regarding these results. These may include, but are not limited to:

1. The patient has already had previous *BRCA* testing in a diagnostic laboratory.
2. The patient has had progressive disease such that *BRCA* status does not change or impact decisions on further treatment options.

It is left to the investigator's discretion whether any of the above, or other circumstances exists, that do not warrant contacting the patient to inform them of the results of the study.

Contact(s) for Further Information/Questions
